# Human Preferences for Conformation Attributes and Head-And-Neck Positions in Horses

**DOI:** 10.1371/journal.pone.0131880

**Published:** 2015-06-30

**Authors:** Georgina L. Caspar, Navneet K. Dhand, Paul D. McGreevy

**Affiliations:** Faculty of Veterinary Science, University of Sydney, Sydney, New South Wales, Australia; Harvard University Faculty of Arts and Sciences, UNITED STATES

## Abstract

Human preferences for certain morphological attributes among domestic animals may be entirely individual or, more generally, may reflect evolutionary pressures that favor certain conformation. Artificial selection for attributes, such as short heads and crested necks of horses, may have functional and welfare implications because there is evidence from other species that skull shape co-varies with behaviour. Crested necks can be accentuated by flexion of the neck, a quality that is often manipulated in photographs vendors use when selling horses. Equine head-and-neck positions acquired through rein tension can compromise welfare. Our investigation was designed to identify conformations and postures that people are attracted to when choosing their ‘ideal’ horse. Participants of an internet survey were asked to rate their preference for horse silhouettes that illustrated three gradations of five variables: facial shape, crest height, ear length, ear position and head-and-neck carriage. There were 1,234 usable responses. The results show that overall preferences are for the intermediate, rather than extreme, morphological choices (p=<0.001). They also indicate that males are 2.5 times less likely to prefer thicker necks rather than the intermediate shape, and 4 times more likely to prefer the thinner neck shape. When compared to the novice participants, experienced participants were 1.9 times more likely to prefer a thicker neck shape than the intermediate neck shape and 2.8 times less likely to prefer a thinner neck shape than the intermediate neck shape. There was overall preference of 93% (n=939) for the category of head carriage ‘In front of the vertical’. However, novice participants were 1.8 times more likely to choose ‘behind the vertical’ than ‘in front of the vertical’. Our results suggest that people prefer a natural head carriage, concave facial profile (dished face), larger ears and thicker necks. From these survey data, it seems that some innate preferences may run counter to horse health and welfare.

## Introduction

As with other domesticated species, the domestic horse (*Equus caballus)* shows a diverse morphology [1,2,3,4]. Over many centuries, horse shapes appear to have been modified to suit different human purposes [5]. The motivation for human preferences has no doubt changed over time with the shifting role of the horse in society [6]. More recently, and in contrast to many livestock species, horse breeding has developed with little requirement for profit [7] and, although horses do not share our living space alongside dogs and cats, they are often described as a ‘companion animal’ [1]. It is therefore unsurprising that an aesthetic appreciation of conformation (morphology) can positively influence financial value [7]. Judgment of non-performance traits can be quite subjective even though some aspects of conformation relate directly to performance [7]. This paper explores human preferences for some elements of equine appearance.

Exploring our preferences for certain types of equine conformation requires consideration of whether humans have an innate preference, regardless of their equestrian knowledge and experience. Wilson [8] coined the term biophilia to describe “the innately emotional affiliation of human beings to other living organisms”. Expanding on this theory, it has been further suggested that environmental factors, such as availability of resources, access to shelter, terrain and lack of hazards, all cause an innately positive response in a human observer due to their tendency to promote survival [9]. Furthermore, numerous studies have identified that people living in urban and industrial societies show a preference for landscapes that incorporate bucolic elements [9]. This preference for open spaces and grassy landscapes suggests perhaps that our agrarian past continues to influence contemporary preferences.

Whether judging a pony in the show-ring, examining a horse as a veterinarian or choosing a racehorse on which to gamble, observers often visually assess equids while standing to one side [1,10]. This strongly implies that the outline of the horse’s body is a core attribute and, unsurprisingly, breed standards reflect a focus on conformation as viewed from the side [11]. In the same vein, McGreevy et al [12] reported that neck flexion is manipulated in advertisements of horses and ponies as riding animals. This suggests that head-and-neck attributes are of particular interest to prospective buyers of horses. The appeal of flexed necks is further supported by recent studies of dressage judges who, despite being chiefly responsible for an assessment of the locomotory activity of the horse as a whole, focus their visual attention preferentially on the cranial half of the horse (including the head, neck and chest) at the expense of attention to the caudal half of the horse [13].

Pedomorphosis resulting from humans favoring youthful-looking animals has been described by Goodwin et al [14] who also showed that particular traits in dogs are associated with behavioral differences. More recently, it has been suggested that, like dogs, some breeds of horses show signs of pedomorphosis [15]. Concave nasal profiles are typical of so-called hotblood morphotypes whereas convex nasal profiles (or Roman nose shapes) are typical of so-called coldblood morphotypes[16]. In horse breeding, small heads and concave nasal profiles (so-called dished faces) have historically been associated with perceived improvements when applied to some of the thicker-set or more heavily boned horse breeds [17]. Examples include the introduction of Arabian bloodlines to modify the New Forest Pony or the Moroccan Barb [3,18]. Conversely, horses with large heads and convex profiles are regularly described as having common or coarse features [19]. This may represent a legacy from the days when work-horses were regarded as inferior in a riding context and when horses with relatively small or dished heads, such as Thoroughbreds, were status symbols [20]. The connection of horses to wealth and aristocracy is as ancient as the connection of horses to warfare [21]. Is it possible therefore that, historically, riding horses were considered a luxury and therefore we persistently favor an animal with pedomorphic features over a placid, draught type?

In considering the possibility of equine pedomorphosis, the appearance of the pinnae is significant because small ears have been reported as a pedomorphic feature [15]. There is an abundance of opinion on what constitutes the ‘ideal’ equine head, small ears being seen as both advantageous to, for example, temperament [22] or to be avoided [23]. Ears pointed forward are anecdotally described as signifying a pleasant demeanor [24], even though this position may simply mean the horse is attending to a stimulus in front of it [1].

Recently, head-and-neck positions have been subject to intense scientific scrutiny because of the current debate about the perceived benefits and disadvantages and compromised welfare of a specific head-and-neck position known as hyperflexion (also known as rollkur, long deep and round training) [25,26,27,28]. Hyperflexion means the horse is ridden with its nasal planum behind the vertical. Current dressage rules require the horse to be ridden with the nose slightly in front of the vertical. However, recent studies have shown that dressage scores are not affected by this flaw [29] and that hyperflexion is associated with conflict behaviors that speak of compromised welfare [30]. McGreevy and Mclean [31] suggest that, far from being seen as a training deficit, this position has become desirable for aesthetic reasons. This important because hyperflexion cannot be achieved without some rein tension that necessarily deteriorates the deceleration response from bit cues [31]. Horses with poor deceleration responses are harder to control, representing a safety risk for the rider [12].

This paper explores human preferences for the characteristics of the equine head and neck. It explores preference for facial profile, crest height, ear length, ear position and head-and-neck carriage.

## Methods

### Questionnaire design and sampling

**Fig 1:**
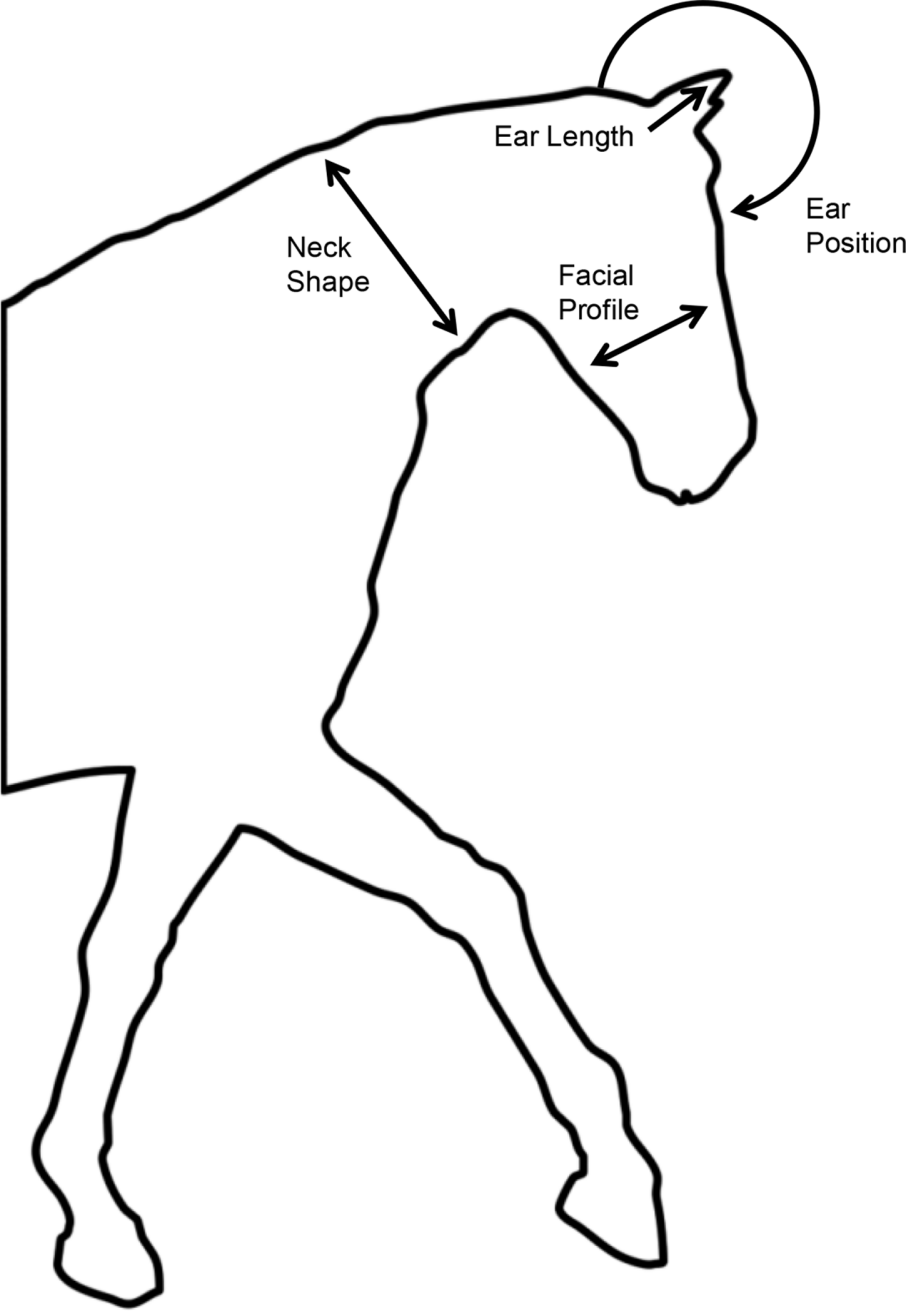
Measurements that appeared at six variants for each attribute presented in random order.

An online questionnaire was designed using the program SurveyMonkey (SurveyMonkey Inc., California, USA, www.surveymonkey.com) to gather information from horse owners and non-horse owners about their ‘ideal horse’. It consisted of nine closed questions. Participants were asked to select what they considered to be a representation of their ‘ideal’ horse. A silhouette of the head and neck of a horse was taken from stock photo footage (Fig 1). This silhouette was altered using Adobe Photoshop (Adobe Systems Pty. Ltd Sydney, NSW, 2000, Australia) to create various versions with different facial profile, ear length, neck shape, ear position, and head-and-neck carriage. Pictures were presented in a random order to participants who were then asked “Which shape is closest to your ideal horse?” and allowed to choose one image.

Advertisements were placed on website forums calling for participants in a “Horse Selection” survey. Forums included Cyberhorse (www.cyberhorse.com.au), Horseyard (www.horseyard.com.au) and Bush Telegraph (www.bushtelegraph.com). A web link was placed on the homepage of the Faculty of Veterinary Science and the Human Animal Research Network at The University of Sydney. Two emails (an initial and a follow-up) with links to the survey were sent directly to Veterinary Science and Animal and Veterinary Bioscience undergraduate students at The University of Sydney’s Faculty of Veterinary Science requesting participation, regardless of whether students considered themselves experienced with horses. Approaches were also made to secretaries of the Australian Campdraft Association, Pony Club Association, Endurance Association, South Australian Dressage Association, Dressage NSW, National Pleasure Horse Association, Victorian Eventers Association and Horse Riding Clubs Association. In addition, 27 National Breed Associations were also emailed to request the participation of members.

This study was conducted under the approval of the University of Sydney Human Research Ethics Committee (approval number: 01-2010/12396). Participants in this study read the following information statement before commencing the survey:

*“Your involvement is strictly confidential. Any research data gathered from the results of the study may be published however no information about you will be used in any way that is identifiable.*

*You can withdraw from the study prior to submitting your completed questionnaire/survey, without affecting your relationship with the researcher(s) or the University of Sydney now or in the future.*

*Being in this study is completely voluntary and you are not under any obligation to consent to complete the questionnaire/survey. Submitting a completed questionnaire/survey is an indication of your consent to participate in the study. Once you have submitted your questionnaire/survey anonymously, your responses cannot be withdrawn.”*

The survey was also spread through social media channels (e.g. Facebook) and participants were asked to encourage others to take part and recruit a large variety of people, both with and without horse riding and handling experience.

### Conformation attributes

#### Facial profile

**Fig 2:**
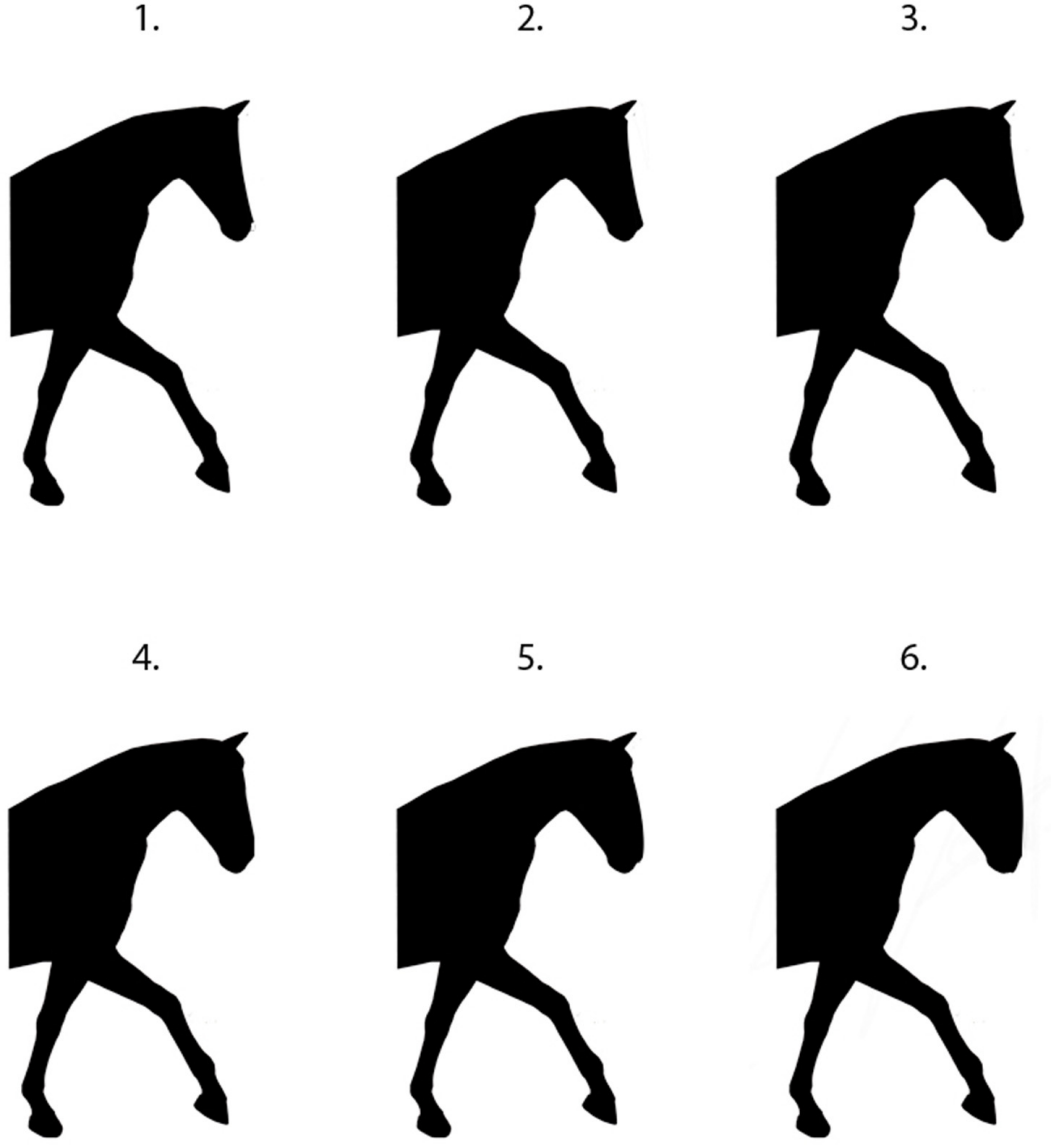
The six facial profile illustrations presented in random order to participants.

Facial profile was altered from the original image by changing the distance from the gullet (which was taken as the junction of the ventral midline with the caudal-most point of the mandible) to the nearest point on the nasal plane (Fig 1). Starting with the original silhouette this metric was varied in percentages as follows: version A: -11.49%, version B: -9%, version C: original, version D: +2.3%, version E: 4.5%, version F: 13.6% (Fig 2)

#### Ear length

Relative ear length was altered by adjusting the proportion of two measurements.

1. The length of the rostral edge of the pinna, and
2. The distance from the tip of the upper lip to the interception of the base of the pinna (Fig 1).

**Fig 3:**
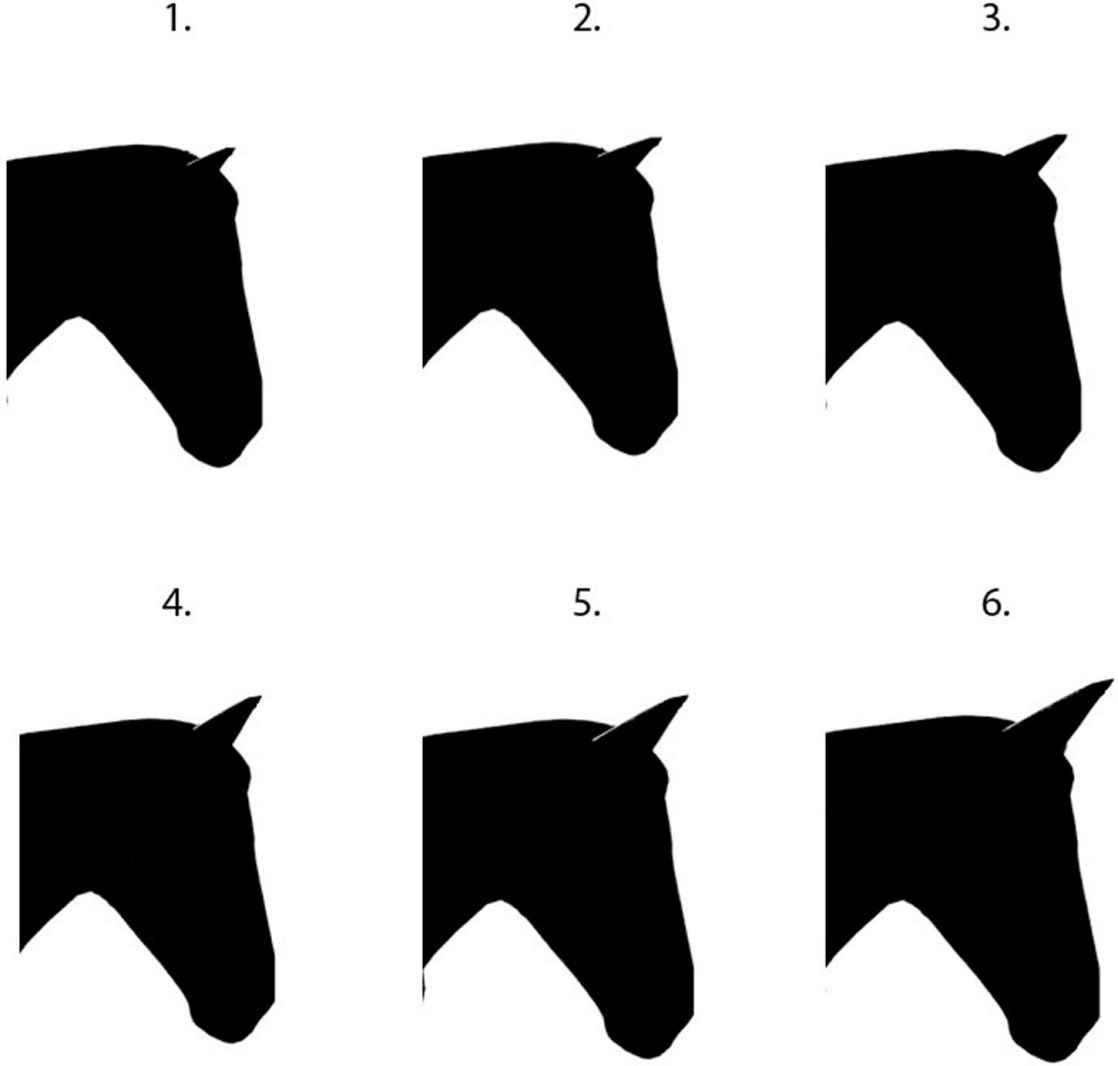
The six ear size illustrations presented in random order to participants.

The percentages were as follows: version A: 9.4%, version B: 14.3%, version C: 16.7%, version D: 20%, version E: 25% and version F: 28.9%. (Fig 3)

#### Neck shape

**Fig 4:**
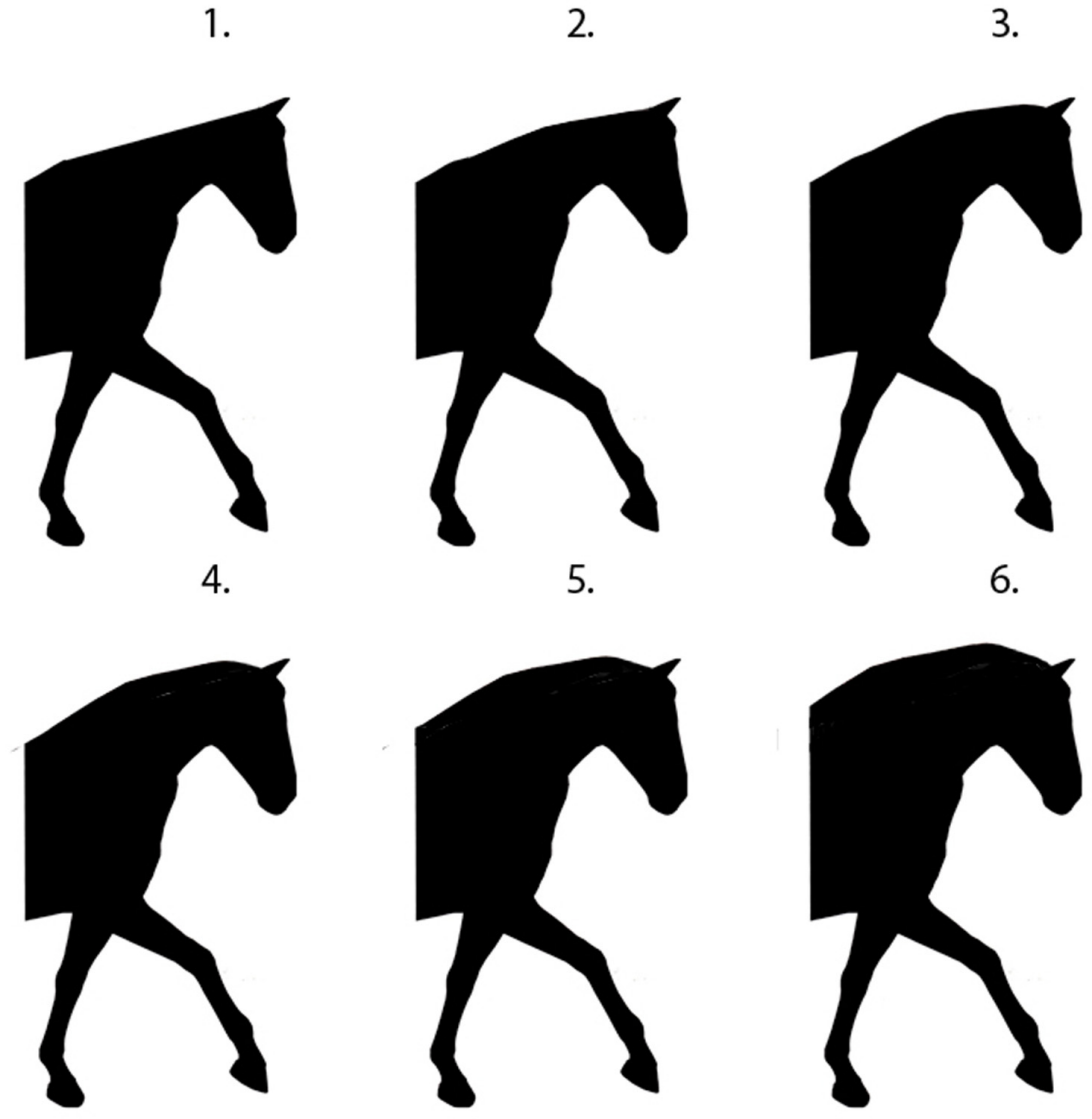
The six neck shape illustrations presented in random order to participants.

Neck shape was altered by changing the width of the neck (which was taken as the distance from the gullet to the closest point on the dorsum of the neck (Fig 1)). Starting with the original silhouette, this metric was varied in percentages, as follows: version A: -14%, version B: -11.6%, version C: original photo, version D: +7%, version E: +16% and version F: 26% (Fig 4)

#### Ear position

**Fig 5:**
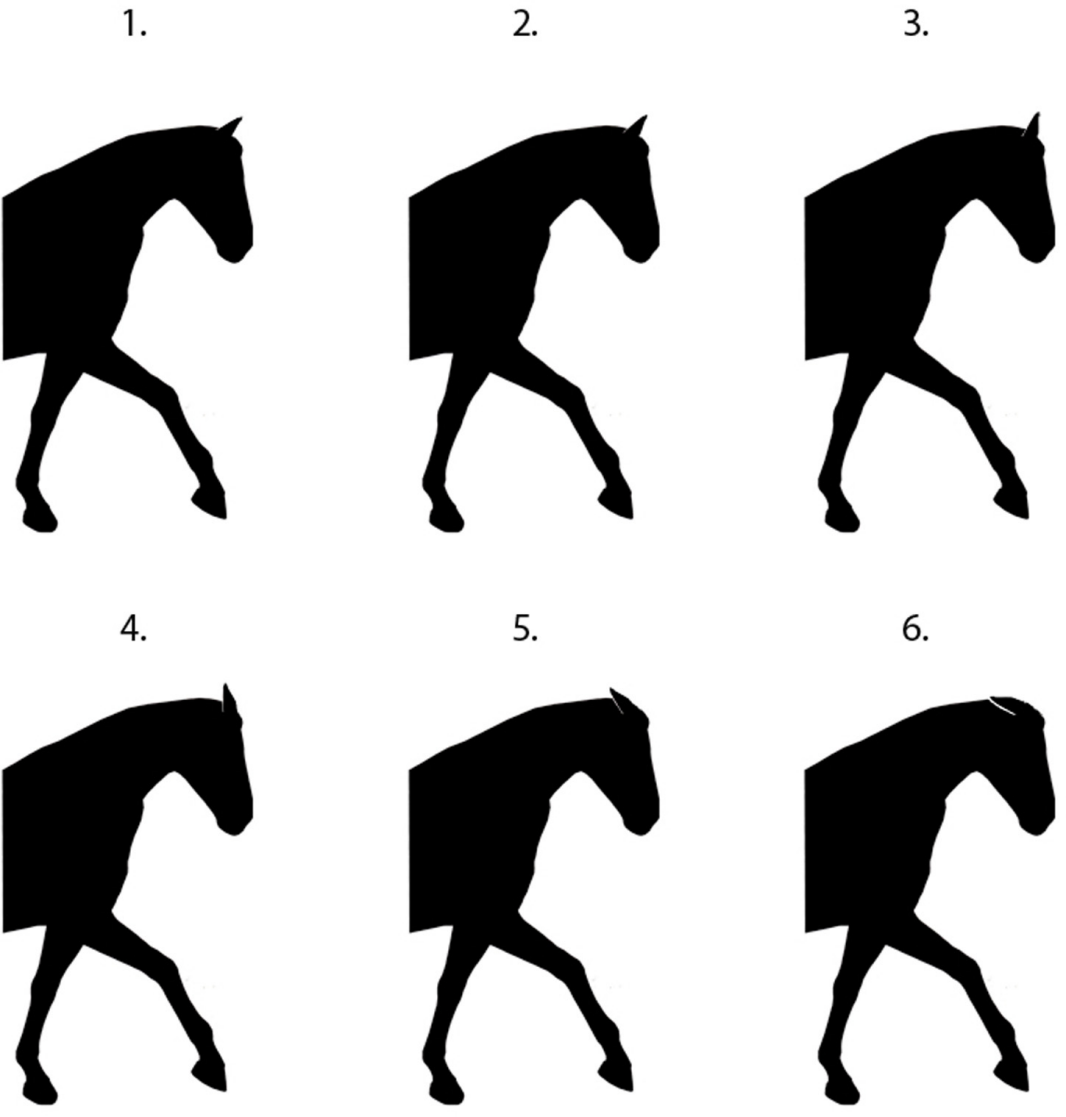
The six ear position illustrations presented in random order to participants.

Ear position was altered by changing the angle of the ear (which was taken as the angle between the rostral border of the pinna and the plane of the front of the head). Starting with the angle at 91.5°, for the forward-pricked ears this metric was varied in gradations as follows: version A: 91.5°, version B: 113°, version C: 137°, version D: 163°, version E: 199°, version F: 226° (Fig 5)

### Head-and-neck carriage

**Fig 6:**
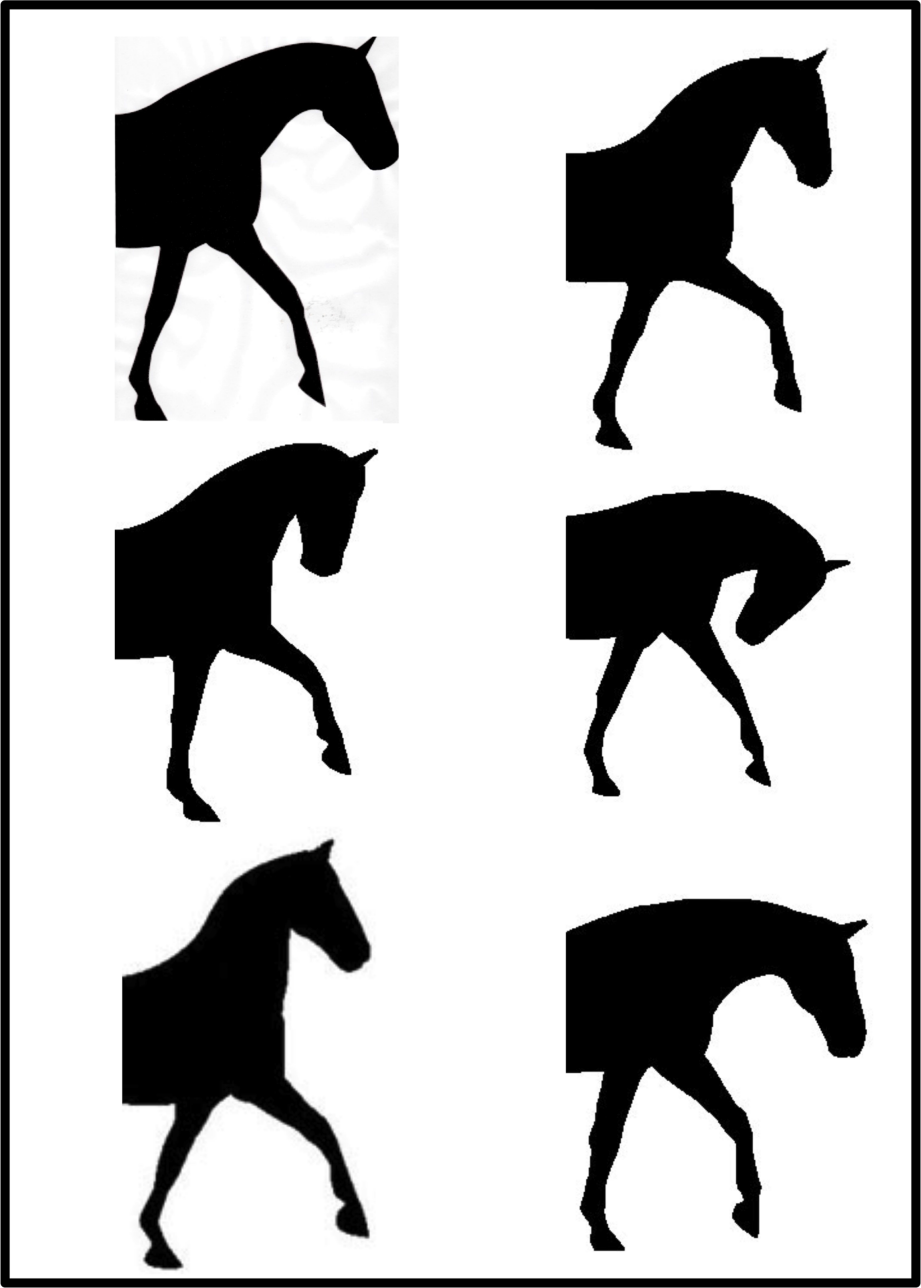
The six head-and-neck positions presented in random order to participants (A-E from L to R).

**Fig 7:**
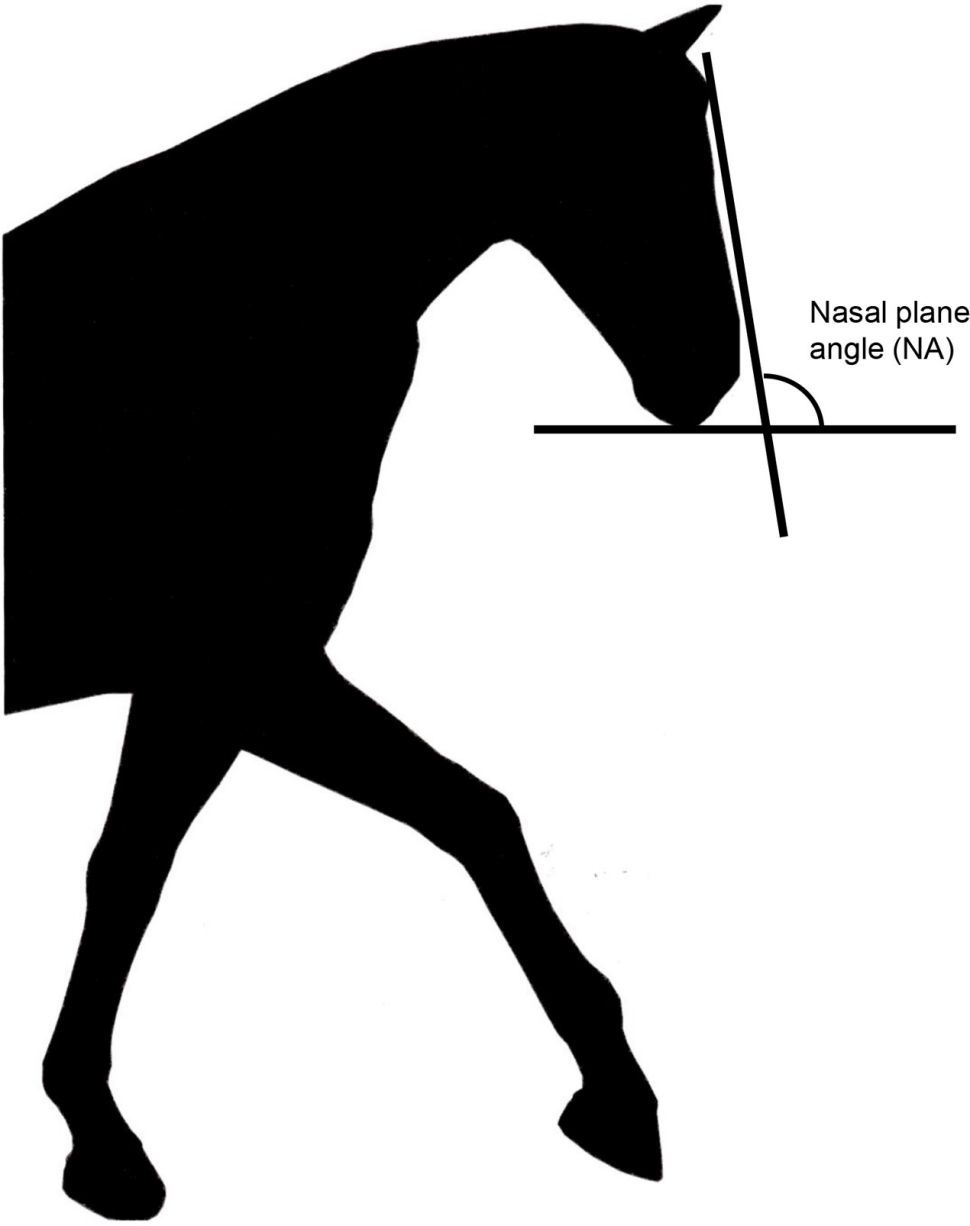
Original silhouette and nasal angle measurement.

Six images were obtained from a previous study that explored neck position and head carriage Alvarez (2006) and altered in Adobe Photoshop (Adobe Systems Incorporated, Sydney, NSW, 2000, Australia) to form silhouettes of the original images (Fig 6). Head-and-neck position number 5 (HNP 5) from the original graphic was altered to ensure that the ear position of all horse profiles was similar. The nasal planes of horses in these six illustrations were measured by relating the angle of the nasal plane to the horizontal plane (Fig 7). The angle formed between the nasal plane and the horizontal plane was labelled the nasal angle (NA). The NA images were presented in random order. The measurements for these figures were as follows: ‘behind the vertical’ (HPN4–43°, HPN3–15°) and ‘in front of the vertical’ (HPN2 10°, HPN1 12°, HPN6 20°, HPN3 28°).

### Demographic characteristics

Three demographic characteristics were considered to evaluate their association, as explanatory variables, with the conformation attributes (above). In the questionnaire the participants were asked about their experience with horses. The possible responses from which they could select were: no experience; casual rider as a child; casual rider as an adult; rider with at least 2 years’ experience and rider with at least 8 years’ experience. Respondents were required to indicate their gender (male or female) and age in years, which was split into four categories: 18–30, 31–45, 46–60 and 61–80 years.

#### Statistical analysis

‘Experience’ was collapsed into two categories: no experience, child rider, casual adult rider or under 2 years’ experience became the ‘Novice’ category and over 8 years’ experience became the ‘Experienced’ category. The facial profile, ear length and neck shape were collapsed into three categories: ‘Hotblood’ shape was represented by versions A and B, ‘Intermediate’ represented by versions B and C and ‘Coldblood’ shape was represented by versions D and E. Ear direction was categorised into ‘Ears forward’ (version A/B), ‘Ears neutral’ (version C/D) and ‘Ears backwards’ (version E/F). The head-and-neck positions were categorised into ‘in front of the vertical’ and ‘Behind the vertical’.

Once the survey was closed, data were managed using Excel 2010 (Microsoft corporation) and statistical analyses were conducted using SAS statistical program (Version 9.3 2002–2010 by SAS Institute Inc., Cary, NC, USA.) and IBM SPSS Statistics Version 21.0. (IBM Corp. IBM SPSS Statistics for Windows, Armonk, NY).

Initial descriptive analyses included creation of frequency tables for demographic variables and conformation attributes. Contingency tables were created to explore any associations between respondents’ demographic variables and their preference for both conformation attributes and head-and-neck positions.

The proportions of respondents preferring a particular conformation attribute were compared using chi-squared tests. Univariable logistic regression analyses were conducted to investigate the unconditional association of demographic characteristics with each of the conformation attributes, using binomial or nominal logistic regression as appropriate. If more than one demographic variable was significant for a conformation attribute, multivariable binominal or nominal logistic regression analyses, were conducted, as appropriate. Interactions between significant demographic variables were then tested and retained in the model. Odds ratios with 95% confidence intervals and p-values were reported. All reported p-values are two-sided.

## Results

1,243 responses were received over a 15-month period (1 March 2012–1 June 2013). Some participants did not answer all the questions. The distribution of missing data appears in Table 1.

**Table 1:** Descriptive statistics for total numbers of respondents that answered each question in the survey.

	Total	FigA	FigB	FigC	FigD	FigE	FigF		Females	Missing	Novice	Experienced	Missing	18–30	31–45	46–60	61–80	Missing
**Facial Profile**	**1017**	**131**	**152**	**298**	**295**	**93**	**48**	**62**	**936**	**19**	**220**	**797**	**0**	**395**	**321**	**238**	**44**	**19**
**Neck Shape**	**1005**	**19**	**33**	**238**	**332**	**242**	**141**	**62**	**936**	**7**	**219**	**786**	**0**	**395**	**321**	**238**	**44**	**7**
**Ear length**	**1004**	**19**	**39**	**128**	**388**	**360**	**70**	**62**	**936**	**6**	**218**	**786**	**0**	**395**	**321**	**238**	**44**	**6**
**Head-and-neck carriage**	**1009**	**286**	**332**	**55**	**15**	**60**	**261**	**62**	**936**	**11**	**219**	**790**	**0**	**395**	**321**	**238**	**44**	**11**
**Ear direction**	**1013**	**66**	**204**	**384**	**269**	**74**	**16**	**62**	**936**	**15**	**220**	**793**	**0**	**394**	**321**	**238**	**44**	**15**

Of the respondents, the majority (94%) were females (Table 2). The median age of the respondents was 35 years for females and 41.5 years for males, with a range between 18 and 80+. The majority of respondents (78%) identified themselves as Experienced (Table 2).

**Table 2:** Descriptive results for demographic explanatory variables (n = 998).

Age of Respondents	Gender		Total
	Female	Male	
18–30	380 (96%)	15 (4%)	395 (40%)
31–45	301 (94%	20 (6%)	321 (32%)
46–60	216 (91%)	22 (9%)	238 (24%)
61–80	39 (89%)	5 (11%)	44 (4%)
**Levels of Experience**			
Novice	188 (87%)	28 (13%	216 (22%)
Experienced	748 (94%	34 (6%)	782 (78%)

### Conformation attributes

Overall, across all morphotypes–when age, experience or gender of respondents was ignored–intermediate types were preferred (all P-values <0.001) (see the last ‘Total’ column in Table 3). Contingency tables of the five outcome variables are shown in Tables 2 and 3 for demographic variables and conformation attributes and head-and-neck positions, respectively.

**Table 3:** Contingency table for variables associated with Conformation attributes and head-and-neck carriage reported by respondents (n = 998) to an online survey.

Variables and categories	Gender		Experience		Age				
	Female	Male	Novice	Experienced	18–30	31–45	45–60	61–80	TOTAL^a^
**Head Shape**									
Hotblood	255(27%)	22 (36%)	50 (23%)	227 (29%)	101 (26%)	89 (28%)	72 (30%)	15 (34%)	283 (28%)
Intermediate	548 (59%)	34 (55%)	136 (63%)	446 (57%)	238 (60%)	192 (60%)	129 (54%)	23 (52%)	593 (58%)
Coldblood	113 (14%)	6(10%)	30 (15%)	109 (14%)	56 (14%)	40 (13%)	37 (16%)	6 (14%)	141 (14%)
**Ear Length**									
Smaller	50 (5%)	7 (11%)	15 (7%)	42 (5%)	22 (6%)	17 (5%)	14 (6%)	4 (9%)	58 (6%)
Intermediate	481 (51%)	32 (52%)	112 (52%)	401 (51%)	209 (53%)	162 (50%)	123 (52%)	19 (43%)	516 (51%)
Larger	405 (43%)	23 (37%)	89 (41%)	339 (43%)	164 (42%)	142 (44%)	101 (42%)	21 (48%)	430 (43%)
**Neck Shape**									
Hotblood	39 (4%)	12 (19%)	26 (12%)	25 (3%)	25 (6%)	14 (4%)	8 (3%)	4 (9%)	52 (5%)
Intermediate	527 (56%)	40 (65%)	137 (63%)	430 (55%)	211 (53%)	185 (58%)	146 (61%)	25 (57%)	570 (57%)
Coldblood	370 (39%)	10 (16%)	53 (25%)	327 (42%)	159 (40%)	122 (38%)	84 (35%)	15 (34%)	383 (38%)
**Ear Direction**									
Ears forward	249 (27%	17 (27%	68 (32%)	198 (25%)	107 (27%)	84 (26%)	57 (24%)	18 (41%)	270 (27%)
Ears neutral	603 (64%)	40 (65%)	126 (58%)	517 (66%)	254 (64%)	212 (66%)	158 (66%)	19 (43%)	653 (64%)
Ears backwards	84 (9%)	5 (8%)	22 (10%)	67 (8%)	34 (9%)	25 (8%)	23 (10%)	7 (16%)	90 (9%)
**Head-and-neck carriage**									
In front of the vertical	872 (93)	58 (94%)	194 (89%)	736 (94%)	351 (89%)	305 (95%)	230 (97%)	44 (100%)	939 (93%)
Behind the vertical	64 (4%)	4 (6%)	22 (11%)	46 (6%)	44 (11%)	16 (5%)	8 (3%)	0	70 (7%)

^a^These proportions for facial profile, ear length, neckshape and ear direction were significantly different. The intermediate shape was preferred (p<0.001)

#### Facial profile and ear length

There were significant differences between respondents choice of the three variables. The maximum proportion of people chose the intermediate shape for facial profile (58% n = 593). However, the hotblood profile was chosen by 28% (n = 283) of participants compared to 14% (n = 141) choosing the coldblood profile. Whilst 51% chose an intermediate ear length, larger ears were chosen by 43% (n = 430) of participants over the smaller options, that were chosen by 6% (n = 58) in this study.

#### Neck shape

The maximum proportion of people (57% n = 570) prefer the intermediate shape. Outside of the intermediate shape, 38% (n = 383) preferred a thicker neck shape. Among respondents showing this preference, 39% (n = 327) were female and 42% (n = 370) were experienced.

Females were significantly more likely to select thicker necks (Odds Ratio (OR) 2.8; 95% Confidence Interval (CI) 0.19 to 8.20; P <0.001) than intermediate necks and less likely to choose thinner necks (0.36; 0.17 to 0.70; P<0.001). In other words, females were 2.8 times more likely to choose a thicker neck, compared to males and about 3 times less likely to choose a thinner neck. Clearly with the number of males we need to interpret these results with caution.

Experienced respondents were more likely to choose a thicker neck (OR 2.0; 95% CI: 1.4 to 2.8; P <0.001) than an intermediate one and less likely to choose a thinner neck (OR 0.31 95% CI: 0.17 to 0.55; P <0.001) than an intermediate one.

A multivariable model was built to evaluate associations of gender and experience with neck shape and both of these variables were significant in the final model.

The results suggest that males are 2.5 times less likely to prefer thicker necks (0.40, 95%CI, 0.18, 0.78) rather than the intermediate shape, and 4 times more likely to prefer the thinner neck shape (3.22, 95%CI 1.48, 6.63). When compared to the novice respondents, experienced respondents were 1.9 times more likely to prefer a thicker neck shape than the intermediate neck shape (1.88, 95% CI: 1.33, 2.68) and 2.8 times less likely to prefer a thinner neck shape than the intermediate neck shape (0.35, 95% CI 0.19, 0.64). So, even allowing for gender differences, our experienced respondents preferred thicker necks.

#### Ear direction

The maximum proportion of respondents (64%) chose the ‘ears neutral’ position (n = 653). Outside of the intermediate preferences, 27% (n = 270) preferred the ‘ears forward’ position in comparison to 9% (n = 90) who chose ‘ears backwards’. Across the age groups, older respondents were more likely to prefer an ‘ears forward’ position (n = 18, 41%) rather than the ‘ears backward’ position. There were few differences in preferences between the other explanatory variables of gender and experience.

#### Head-and-neck carriage

Among all respondents there was a preference of 93% (n = 939) for the category of head-and-neck carriage ‘In front of the vertical’ compared to 7% (n = 70) of ‘behind the vertical’ (P =) <0.001 (Fig 1).

Based on a Likelihood Chi-Squared test, there was a significant association between experience and preferred head-and-neck carriage (p = 0.028). Results indicate that novice respondents were 1.8 times more likely to choose ‘behind the vertical’ (Odds Ratio (OR) 1.8, 95% CI: 1.05 to 3.06) than ‘in front of the vertical’.

None of the respondents in the ‘80+’ category chose ‘behind the vertical’, so the logistic regression model could not be fitted. Instead, the Fishers Exact Test was used. This showed a significant association between head-and-neck carriage and age (p = <0.005). Younger respondents were more likely to choose ‘behind the vertical’. Within the current dataset, younger respondents were more likely to prefer head carriage that was ‘behind the vertical’, a finding that merits further scientific scrutiny. It would be especially interesting to explore interactions between age and dressage competition experience on this preference.

There were no other significant differences between outcomes and explanatory variables.

## Discussion

This study explored conformation attributes and head-and-neck positions that people prefer in horses, and how these change with experience, age or gender. Participation in this study was voluntary and, due to the nature of viral spread, determining whether it was a true representation of the general population is difficult. We were particularly interested in discovering what representatives of the general population preferred in their ideal horse. However, the numbers of respondents with substantial horse experience far outweighed those with little or no experience, so future studies would benefit from recruiting more inexperienced respondents. Similarly, female respondents were over-represented in the current sample by 88%. That said, comparable equine studies with voluntary participants have noted a similar gender bias [32,33]. The distribution of males and females is possibly reflective of participation in equestrian sports, that show a high ratio of females to males [34].

There was a strong overall preference for intermediate morphotypes and postural attributes. This meant that the preference variations between age, experience and gender were small. However, some features, specifically neck shape and head-and-neck carriage were highly significant.

Although the majority of respondents chose the ‘neutral’ outlines, the preferences other than neutral were for thicker necks, concave facial profiles and longer ears. These trends are not surprising given the current preponderance of sport horses with such features being marketed in the equestrian media [35]. Further study could explore the relationship between experience, discipline and preferences. However, equestrian respondents may be more likely to be exposed to the modern sport horse, whereas those with less equestrian experience may arguably be exposed to televised images of racehorses and thus be more familiar with the Thoroughbred shape and its gracile proportions.

There is some evidence that, in both horses and dogs, skull shape is associated with differences in the arrangement of neural tissue, specifically ganglion cell distribution, in the retina [36,37]. This suggests that short-skulled dogs may perceive stimuli differently to long-skulled dogs. Furthermore, there is recent evidence, again in dogs, that head shape co-varies with behavior [2]. Some data suggest that Thoroughbreds, with their typically shorter than average skulls [38] are more anxious and excitable [39]. In some contexts, such as racing and showing, such reactivity is desirable even though it may compromise rider safety. This suggests that, outside equestrian disciplines such as racing that favor reactivity, having a relatively pedomorphic head may be desirable for aesthetic rather than functional reasons. As it happens, any tendency to select for small heads, at the same time as long legs, may also inadvertently contribute to some performance breeds being more prone to locomotor asymmetry; an attribute that can compromise athletic ability [40].

Prior to domestication, horses were primarily valued as a source of food [1]. Cave paintings, such as those seen in Lascaux, France depict heavily crested and large-bodied equidae [1]. These images suggest that we may have learned to favor well-nourished horses. It is possible that this persists as an innate preference, regardless of whether we are horse owners, riders or just observers. For most respondents in the current study, the ideal horse has a thick neck. In addition, there is some evidence that, despite awareness of the health detriments associated with equine obesity, some knowledgeable horse people deliberately overfeed their horses to produce the rounded body shape that is often rewarded by judges in the show-ring [31]. Within the current data, experienced females, significantly more than experienced males, preferred a thicker neck. These results do need to be interpreted with caution due to the low numbers of male respondents and the possibility that experienced respondents came from a specific equestrian discipline. That said, the preferences reported here may explain some of the breeding and training practices that prevail within some popular equestrian disciplines.

Horses displaying excessive flexion of the neck appear to have a larger crest than when they are flexing their neck naturally [12,41]. Perhaps it is the neck’s flexion, rather than its thickness, that humans find particularly appealing. Such flexed necks are found in artworks of many cultures including Japanese, Chinese and European [42]. Depictions of war horses, royal horses, wild horses being contained and horses engaged in sport are almost always represented with an unnaturally arched neck [43]. From a nutritional perspective, horses with thicker necks are generally fatter. So, returning to the preceding anthropological discussion, this provides a link that supports the biophilia theory because, as carcasses, these horses would be more energy dense and therefore more desirable to hunters. Equally, those seeking a strong draft animal would also favour this phenotype.

In several equestrian disciplines, excessive flexion of the poll (i.e., atlanto-occipital joint and cervical vertebrae) is a desired posture that horses are specifically trained to perform. For example, in dressage and showing, there is usually a competitive advantage in the horse maintaining such a position. Breeds frequently used for showing (under saddle and in-hand) and for dressage have been selectively bred for a well-muscled, arched neck [44]. So, it is possible we have revealed a bias that reflects a particular type of equestrian experience. However, the current study did not explore the equestrian disciplines with which the equestrian respondents most closely identified, because the primary aim was to compare preferences between horse owners and non-horse owners.

The preference reported here for natural carriage rather than hyperflexed positions suggests that, regardless of horse experience, humans recognize that horses carrying their head-and-neck behind the vertical are not ‘ideal’. This is surprising given that, within the world of horse buying and selling, most horses are depicted in these positions [12,45]. Flexing the horses’ necks restricts their vision and may make them more compliant [46], possibly by sparing them sight of objects to the fore that they might otherwise avoid or even flee from [12]. This could have led to a perception among riders that hyperflexed horses are easier to control. While it may be so in the short term, relentless bit pressure actually deteriorates deceleration responses ([12]), an outcome that contributes to concerning statistics on horse rider safety [47].

## Conclusion

The current study reveals aesthetic preferences that have no relationship to function and that may even run counter to horse health and rider safety. Outside of the 'neutral' outlines, the preferences were for thicker necks, concave facial profiles and longer ears. The apparent appeal of thicker necks is important because some training techniques that make horses’ necks appear convex, particularly hyperflexion, have been shown to compromise horse welfare and may jeopardize rider safety. Further research should explore the origins of these preferences and whether they influence breeding and riding decisions.
